# Single-cell transcriptional changes of artemisinin-sensitive K13^C580^ and artemisinin-resistant K13^580Y^ *Plasmodium falciparum* upon dihydroartemisinin exposure

**DOI:** 10.3389/fcimb.2026.1816105

**Published:** 2026-07-28

**Authors:** Sean V. Connelly, Clark Cunningham, Ian Petrow, Nazrin Rustamzade, Jenna Zuromski, Deborah M. Sipolski, Christian P. Nixon, Jonathan D. Kurtis, Jonathan J. Juliano, Jeffrey A. Bailey, Cliff I. Oduor

**Affiliations:** 1 Division of Infectious Diseases, Department of Medicine, University of North Carolina, Chapel Hill, NC, United States; 2 Department of Pathology and Laboratory Medicine, Brown University, Providence, RI, United States; 3 Center for International Health Research, Rhode Island Hospital, Providence, RI, United States; 4 Curriculum in Genetics and Molecular Biology, School of Medicine, University of North Carolina, Chapel Hill, NC, United States; 5 Department of Epidemiology, Gillings School of Global Public Health, University of North Carolina, Chapel Hill, NC, United States; 6 Institute for Global Health and Infectious Diseases, University of North Carolina, Chapel Hill, NC, United States

**Keywords:** artemisinin, dihydroartemisinin, drug resistance, Kelch13, *Plasmodium falciparum*, single-cell RNA-seq (scRNA-seq), transcriptome

## Abstract

Artemisinin-based therapies have been central to malaria control, but partial resistance in *Plasmodium falciparum*, driven by mutations in the Kelch13 (K13) protein, threatens these gains. To investigate the molecular basis of this resistance, we applied single-cell RNA sequencing to isogenic parasite lines, K13 wild-type (K13^C580^) and the artemisinin-resistant mutant (K13^580Y^), following a 6h pulse of dihydroartemisinin (DHA). This approach enabled high-resolution profiling across intraerythrocytic stages. Both lines exhibited stage-specific transcriptional responses, with pronounced changes in ring and trophozoite stages. Using Manifold Enhancement of Latent Dimensions (MELD), a computational framework for quantifying transcriptional perturbation, DHA treatment induces stage-specific differences in protein export and metabolic pathways in K13^C580^ and K13^580Y^ parasites, relating to an altered metabolic stress response state. GARP, a potential therapeutic target, was highly differentially expressed in untreated ring stages of K13^580Y^ and K13^C580^. Functional assays confirmed that anti-GARP antibodies retained efficacy against K13^580Y^, supporting its potential as a therapeutic target. These findings provide a comprehensive view of the cellular responses related to artemisinin partial resistance, identify molecular features of pathogenesis, and highlight surface proteins like GARP as promising intervention targets. This work underscores the power of single-cell approaches to dissect drug responses and guide strategies to overcome resistant parasites.

## Introduction

Malaria, caused mainly by the species *Plasmodium falciparum*, accounted for an estimated 610,000 deaths globally in 2024 (World Health Organization, 2025a). The World Health Organization recommends artemisinin-based combination therapies (ACTs) to treat uncomplicated falciparum malaria. ACTs rely on the fast-acting properties of the ART derivative to rapidly clear parasites, while the longer-acting partner drug clears residual parasites. This combination reduces the frequent observation of recrudescent infections when ART derivatives are used alone (Teuscher et al., 2010). Parasites have evolved ART partial resistance (ART-R), which first emerged in western Cambodia and spread throughout the Greater Mekong Subregion in Southeast Asia (Noedl et al., 2008; Imwong et al., 2020). ART-R has now emerged in Africa and is spreading through the continent, endangering ACT efficacy (Ashley et al., 2014; Tacoli et al., 2016; Balikagala et al., 2021; Dimbu et al., 2021; Ebong et al., 2021; Gansané et al., 2021; Moriarty et al., 2021; Conrad et al., 2023; Fola et al., 2023; Juliano et al., 2024; Connelly et al., 2025). ART-R manifests as a decreased susceptibility of ring-stage parasites to ART. Clinical ART-R is defined as parasite clearance half-life of greater than five hours (Dondorp et al., 2009). Increased parasite survival due to ART-R risks the development of partner drug resistance (Björkman et al., 2024). Given Africa suffers the vast majority of disease burden, a public health disaster could arise if ART-R spreads across the continent (Dhorda et al., 2024).

ART-R is due to nonsynonymous mutations in the ß-propeller domain of the Kelch13 (K13) protein (Ariey et al., 2014; Ashley et al., 2014; Straimer et al., 2015). The identification of *K13* (*PF3D7_1343700*) gene mutations as a marker of resistance has provided the impetus for worldwide surveillance to catalog, track, and analyze the distribution of *K13* mutations in malaria-endemic regions. Over 100 different *K13* mutations have been identified (Ménard et al., 2016; MalariaGEN Plasmodium falciparum Community Project, 2016), with thirteen considered validated resistance markers and ten considered candidate resistance markers (World Health Organization, 2025b). The K13^580Y^ mutation predominates in Southeast Asia (Imwong et al., 2020). In Eastern Africa, the mutations K13^561H,675V,469Y,622I^ have emerged and are now found in multiple countries (Ishengoma et al., 2024). Validated *K13* mutants exhibit about tenfold higher residual viability than their genetically matched wild-type parasites when exposed to dihydroartemisinin (DHA) at the early ring stage (Straimer et al., 2015).

A number of studies have examined the complex molecular response to understand the underpinnings of survival. Different *K13* mutations have been investigated through gene editing and protein localization experiments (Straimer et al., 2015; Yang et al., 2019; Birnbaum et al., 2020; Stokes et al., 2021). While most studies focus on the treatment response of the early ring stage, few studies have focused on the effect of ART treatment over asexual stages *in vitro.* A study of stage-specific artemisinin response correlated fluctuations in drug sensitivity to hemoglobin digestion (Klonis et al., 2013). Further, stage-specific gene expression changes were identified in artemisinin-resistant isolates from Southeast Asia, with rings and trophozoites showing reduced expression of metabolic pathways and schizonts showing increased expression of protein metabolism pathways (Mok et al., 2011). Single-cell RNA sequencing (scRNAseq) allows for the investigation of stage-specific gene expression dynamics in ART-R and sensitive lines. scRNAseq has been utilized in multiple cell atlas efforts across malaria species (Howick et al., 2019; Dogga et al., 2024), but its utility to study gene expression response to treatment perturbations has not been fully harnessed (Nötzel and Kafsack, 2022).

In this study, we explored the single-cell transcriptomic profile of *P. falciparum* isogenic lines K13^580Y^ (MRA-1251) and K13^C580^ (MRA-1250) following DHA exposure using a well-based single-cell capture platform, SeqWell (Gierahn et al., 2017; Aicher et al., 2019; Hughes et al., 2020). We focused on the stage-specific transcriptional effects of DHA treatment on the ring and trophozoite *Plasmodium* cell cycle stages. We observed parasite stage specific gene expression profiles and also potential vulnerabilities of the parasite’s response to DHA treatment, such as elevated GARP surface protein expression, that can be a logical therapeutic target for effective combination therapies in the face of ART-R.

## Methods

### Parasite lines and culture

The strains of parasite lines used in this study were MRA-1250 (K13^C580^), an artemisinin-susceptible fast-clearing Cambodian *P. falciparum* isolate, and MRA-1251 (K13^580Y^), a Cambodian *P. falciparum* isolate that features a single nucleotide substitution leading to a C580Y amino acid change (MR4, BEI Resources, Manassas, VA). The *P. falciparum* parasites were maintained at 3% hematocrit in human O^+^ RBCs and *P. falciparum* culture media comprising RPMI 1640 (Thermo Fisher Scientific) supplemented with 0.5% (w/v) Albumax II, 50 mg/L hypoxanthine, 0.225% NaHCO_3_, 25 mM HEPES, and 10 mg/L gentamycin. Parasites were cultured at 37°C in 5% O_2_, 5% CO_2_, and 90% N_2_ (Schuster, 2002). The parasite lines were cultured for 48h before exposing them to DHA and DMSO.

### Drug susceptibility assays

**Figure 1:**
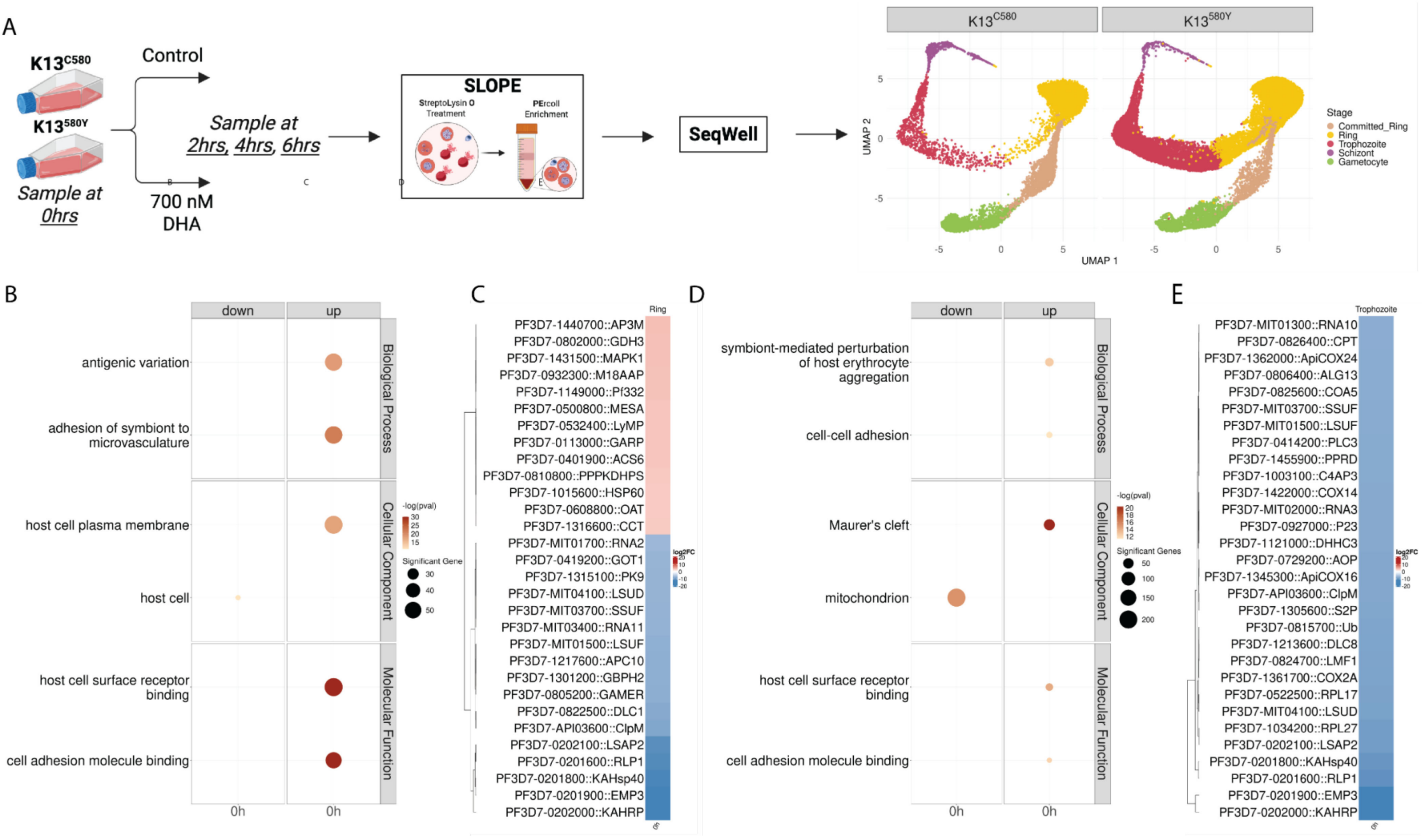
K13^580Y^ versus K13^C580^ rings and trophozoites showed upregulation of parasite-erythrocyte Interaction pathways, with downregulation of mitochondrial and apicoplast genes. **(A)** The experimental workflow is shown, concluding in the UMAP embedding of the final malaria stage annotation over all datasets (Brown: Sexually Committed Ring, Yellow: Ring, Red: Trophozoite, Purple: Schizont, Green: Gametocyte). The graph is separated by strain (left: K13^C580^, right: K13^580Y^). Baseline differential expression was performed. The top 2 significant Gene Ontology (GO) enrichment terms (topGO *p* < 0.01) in each category between K13^580Y^ versus K13^C580^ rings **(B)** and trophozoites **(D)** are plotted, with the number of significant genes that support that GO Term being the size of the circles and the color being the magnitude of the negative log of the topGO *p*-value. A heatmap of the 30 differentially expressed (DE) annotated genes by absolute log2FC between K13^580Y^ versus K13^C580^ rings **(C)** and trophozoites **(E)** are plotted. An asterisk indicates genes that were significantly differentially expressed at an absolute log_2_FC > 1 and a BH-adjusted *p*-value < 0.001. Red boxes are upregulated genes, white boxes indicate genes that were not differentially expressed, and blue boxes are downregulated genes. Created in BioRender. Connelly, S (2026). https://BioRender.com/40clixs.

DHA stocks were made in dimethyl sulfoxide (DMSO), and aliquots were stored at −20°C. All drug assays were conducted such that the final DMSO concentration was <0.5%. K13^C580^ and K13^580Y^ cell cultures were grown over 48h. Unsynchronized parasite cultures were used to profile the transcriptional response to DHA across the intraerythrocytic lifecycle, rather than limiting the analysis to one developmental stage or imposing synchronization pressures that could alter gene expression. We prepared one biological replicate per strain. For each parasite line, seven independently prepared single-cell libraries were generated and analyzed, consisting of one baseline sample collected at 0h before treatment, three DMSO control samples collected at 2h, 4h, and 6h, and three DHA-treated samples collected at 2h, 4h, and 6h. Thus, the integrated dataset used for **Figure 1A** included 14 single-cell libraries in total, seven from K13^C580^ and seven from K13^580Y^. Each library was processed independently through parasite enrichment, SeqWell library preparation, sequencing, read processing, quality control, and initial sample-level analysis before integration.

### Enrichment of parasite-infected RBCs

To prepare the scRNAseq libraries, we enriched the parasite infected RBCs to increase the chances of loading and capturing mRNA transcripts from infected RBCs. Our unsynchronized parasite cultures were enriched using SLOPE as previously described (Brown et al., 2020) with some modifications. Briefly, erythrocyte density was measured using a Cellometer Auto T4 (Nexcelom Biosciences, Lawrence, MA), and cell density was adjusted to 2 × 10^9^ erythrocytes/mL. The 22.5U of the lytic agent streptolysin-O (SLO) per 50 µl RBCs was added in a ratio of two parts SLO solution to five parts erythrocytes. Samples were mixed well by pipetting and incubated at room temperature for 6 min. Ten volumes of 1× PBS or media (RPMI 1640 HEPES) were added and cells were centrifuged at 2,500 × *g* for 3 min. After removal of the supernatant, cells were washed twice more with 1× PBS. Following SLO lysis, cells suspended in 1× PBS were layered onto a 60% Percoll gradient (Sigma Aldrich, St. Louis, MO) and centrifuged at 1,500 × *g* for 10 min. The top layer of Percoll was discarded while the lower cell pellet, rich in infected erythrocytes, was collected and washed twice with 1× PBS and later suspended in 200 µl RPMI to be loaded into the SeqWell arrays.

### Single-cell library preparation and sequencing

To capture single-cell transcriptomes, we utilized SeqWell, a massively parallel, low-input scRNAseq platform (Gierahn et al., 2017; Aicher et al., 2019; Hughes et al., 2020). Details of array loading, library preparation, and sequencing are provided in the **Supplementary Methods**. Raw read data and processed UMI count matrices from K13^C580^ and K13^580Y^ scRNAseq experiments have been deposited and are publicly available through the SRA database (BioProject ID: PRJNA1049964).

### Single-cell transcriptome analysis

Raw reads were processed using version 3.0.2 of the Drop-seq pipeline (Macosko et al., 2015), according to the ‘Drop-seq Alignment Cookbook’, both found at http://mccarrolllab.com/dropseq/. Demultiplexed FASTQs were aligned to a concatenated human (Gencode v48) and *P. falciparum* 3D7 reference genome (PlasmoDB v68) using the STAR aligner. Any reads aligned to human chromosomes were filtered out with a custom script (01_read_filter_human.py) (**Supplementary Figure 1**). Individual reads were tagged with a 12 bp barcode and 9 bp unique molecular identifier (UMI) contained in Read 1 of each sequencing fragment. Following alignment, reads were grouped by the 12 bp cell barcodes and subsequently collapsed by the 9 bp UMI to generate a gene expression count matrix. Gene expression scRNAseq count matrices from K13^C580^ and K13^580Y^ cell line experiments (*n* = 7 per *Pf* cell line) were analyzed and visualized in R (v4.5.2) using the Seurat package (v5.3.1) (Butler et al., 2018).

### Quality control filtering

During quality control filtering, cells with <300 genes, <500 UMIs, and >20% mitochondrial gene expression were removed (**Supplementary Figures 2**, **S3**). Genes were retained if they were expressed in at least 10 cells or expressed in at least 1% of all cells (**Supplementary Figures 4**, **S5**). Thresholds were chosen based on the distributions present in the dataset, balancing removal of low-quality cells while retaining biologically meaningful parasite transcriptional profiles.

### Cell annotation pipeline

The annotation strategy is outlined in **Supplementary Methods** and **Supplementary Figure S6**. Each individual sample underwent doublet detection with scrublet (v0.2.3) and doubletFinder (v2.0.6) (**Supplementary Figures S7**, **S8**). The expected percentage of doublets was estimated to be 2.37% based on previously published SeqWell experiments under comparable loading conditions (Hughes et al., 2020). In Scrublet, a doublet detection threshold of 0.13 was used over all datasets. In doubletFinder, a per-dataset threshold was set using the strategy outlined in the Demuxafy package (Neavin et al., 2024). Any cells that were not present in the developmental trajectory were classified as doublets (**Supplementary Figures S9**, **S10**). After rigorous quality control using doublet detection software, cell filtering and gene filtering, each dataset was initially processed independently (normalization with SCTransform (Hafemeister and Satija, 2019), clustering, and annotation), followed by integration using Harmony (Korsunsky et al., 2019) to align shared transcriptional structure across datasets while mitigating batch-driven effects. Additionally, we visualized marker gene expression in the clusters, correlated our data to multiple bulk RNA sequencing datasets (Painter et al., 2018; Reers et al., 2023), and mapped our cells to multiple single-cell atlases of malaria development to verify cell annotation (**Supplementary Figures S11**–**S13**) (Howick et al., 2019; Dogga et al., 2024). The stage distribution per timepoint and strain is shown in **Supplementary Figure S14, Table S1**.

### Pseudotime

Pseudotime values and weights were calculated using slingshot over the asexual stages (Street et al., 2018) (**Supplementary Figure S15**).

### Differential expression

Two different strategies for differential expression (DE) were performed: one comparing K13^580Y^ versus K13^C580^ parasites at baseline (Baseline Differential Expression) or comparing DHA- versus DMSO-treated cells within each strain using MELD (Fine-Grained Differential Expression) (**Supplementary Figure S16**). Applying the MELD algorithm and performing differential expression (DE) on our data is detailed in the **Supplementary Methods**. The Potential of Heat diffusion for Affinity-based Transition Embedding (PHATE) embedding used in MELD is shown in **Supplementary Figure S17** (Moon et al., 2019; Burkhardt et al., 2021). The parameter optimization results for MELD are shown in **Supplementary Figure S18**. All DE results across stages are in **Supplementary Table S2**. DHA likelihoods were plotted over pseudotime to confirm that DHA-treated cells exhibited higher DHA likelihood scores than DMSO-treated control cells (**Supplementary Figure S19**). Afterwards, Vertex Frequency Clustering (VFC) was utilized to isolate cells that were most or least perturbed by DHA treatment for differential expression (DE) (**Supplementary Figures S20**–**S24**). DE using VFC clusters resulted in similar results to comparing treatment versus control within each stage (**Supplementary Figures S25**–**S27**). Statistical thresholds at a BH-adjusted *p*-value < 0.001 and an absolute log_2_FC > 1 were implemented in all DE analyses with MAST. For heatmap visualization, hypervariable genes were filtered due to their overwhelming signal in the DE results.

### Gene ontology analysis

Using the significant differentially expressed genes (DEGs), GO analysis was performed with pfGO (v2.1, https://github.com/oberstal/pfGO), an R wrapper for topGO (Alexa and Rahnenfuhrer, 2010). GO term enrichment was performed similarly to previous studies (Simmons et al., 2023), with the output DE results from either baseline differential expression or fine-grained differential expression filtered to DEGs with BH-adjusted *p*-values < 0.001 and categorized as upregulated (log_2_FC > 1) or downregulated (log_2_FC < −1). For each timepoint and in each category, GO term enrichment was performed using a Fisher test with the topGO weight01 algorithm. All GO enrichment results across stages are in **Supplementary Tables S3**, **S4**.

### Growth inhibition assay on K13^C580^ and K13^580Y^

K13^C580^ and K13^580Y^ cell lines were synchronized to the ring stage by treatment with 5% sorbitol for at least two successive replication cycles and cultured to the ring stage. Parasites were diluted with O^+^ RBCs to 0.4% parasitemia and 2% hematocrit in incomplete media (RPMI 1640 medium containing 25 mM HEPES and 2.0 g/L sodium bicarbonate) supplemented with 0.5% AlbuMax II, 367.35 µM hypoxanthine, and 25 µg/ml gentamicin. Polyclonal mouse anti-GARP antiserum (10% v/v) (Raj et al., 2020) or normal mouse sera (10% v/v) was added to the cultures in 96-well round-bottom microtiter plates. After a 72h incubation, cultures were stained with Hoechst nucleic acid stain to quantify parasitemia using a CytoFLEX flow cytometer.

## Results

### Experimental design and baseline differential expression between K13^580Y^ and K13^C580^

To study the transcriptomic profiles of *P. falciparum* cells in response to DHA exposure, we isolated isogenic non-synchronized K13^C580^ and K13^580Y^ parasites after 48h in culture, and profiled the single-cell transcriptome at different timepoints after DHA treatment (**Figure 1A**). The genetic difference between K13^580Y^ and K13^C580^ was verified using molecular inversion probes targeting multiple drug-resistant markers (**Supplementary Methods**; **Supplementary Table S5**). All malaria cell cycle stages annotated in **Figure 1A** were present across timepoints, strains, and treatment conditions (**Supplementary Figure S14**). Rings and trophozoite stages were selected for downstream analysis due to their interpretable and robust perturbation-associated transcriptional patterns in the context of DHA exposure.

We first examined baseline DE prior to treatment (Time 0h, **Figure 1A**). The ring-staged K13^580Y^ compared to K13^C580^ showed upregulation of 400 genes and downregulation of 219 genes. GO analysis detected enrichment of pathways related to parasite-erythrocyte interaction among upregulated genes (**Figures 1B, C**), represented by *GARP* (Almukadi et al., 2019), *FIKK5* (Ward et al., 2004)*, LyMP* (Proellocks et al., 2014)*, MESA* (Coppel et al., 1988), and *Pf332* (Hodder et al., 2009). Folate metabolism (*PPPK-DHPS*) and food vacuole metabolism (*ornithine aminotransferase* (*OAT*) (Gafan et al., 2001) and *HSP60*) were enriched in upregulated genes, while mitochondrial ribosomal RNA genes (*LSUD* and *LSUF*) and apicoplast genes (*ClpM*) were downregulated, compared to K13^C580^ parasites. *K13* and *Kelch13 interacting candidate 2* (*KIC2*) (Birnbaum et al., 2020) were significantly downregulated, while *KIC9* was significantly upregulated in ring-staged K13^580Y^ compared to K13^C580^ (**Supplementary Table S2**).

DE analysis between trophozoite-0stage K13^580Y^ versus K13^C580^ (**Figures 1D, E**) had greater DE differences relative to rings, with 201 and 1118 significantly up- and downregulated genes, respectively. Similar to ring staged parasites, parasite-erythrocyte interaction pathways were enriched in upregulated genes. Metabolic pathways were downregulated, including those in the mitochondria (*COX2A*, *COX14*, and *COA5*) and apicoplast, such as isoprenoid pathway member *1-cys peroxiredoxin* (*AOP*) (Swift et al., 2021) and dolichol pathway component *polyprenol reductase* (*PPRD*) (Zimbres et al., 2020). *KIC3* was significantly downregulated in trophozoite-stage K13^580Y^ compared to K13^C580^ (**Supplementary Table S2**).

### Fine-grained differential expression comparing DHA exposure in K13^C580^ or K13^580Y^ Rings

To quantify DHA perturbation across the malaria stages, the MELD algorithm compares DHA treatment to control over timepoints (see **Supplementary Methods**) (Burkhardt et al., 2021). MELD infers a score per cell that reflects condition-associated transcriptional similarity, named the DHA likelihood. This score ranges from 0 to 1, that is, from less to more transcriptionally similar to DHA-treated cells. Visualization of the distribution of DHA likelihoods across the lifecycle using pseudotime, calculated by slingshot (Street et al., 2018), showed similar responses to DHA treatment in both strains, where there are separate transcriptional states based on treatment condition across the lifecycle (**Supplementary Figure S19**). DHA-treated cells had a higher DHA likelihood than DMSO-treated cells. This aligned with expectations that the DHA-treated cells would have higher DHA likelihood values and be closer to the prototypical DHA-treated state, which is a DHA likelihood value of 1.

When focusing on DE within a stage, we isolated cell populations with the highest DHA likelihood within each timepoint using vertex frequency clustering (VFC) (Burkhardt et al., 2021) (**Supplementary Figures S20**–**S24**). We separated these highly enriched clusters of high DHA likelihood cells (enriched in DHA-treated cells) to compare with clusters of cells with low DHA likelihood (enriched in DMSO-treated cells) to better examine drug effects in rings and trophozoites (**Supplementary Figures S21**, **S22**). VFC allowed for the isolation of cells prototypical for the effects of the DHA or DMSO treatment conditions. Direct DE results without MELD were comparable per stage (**Supplementary Figures S25**–**S27**).

**Figure 2:**
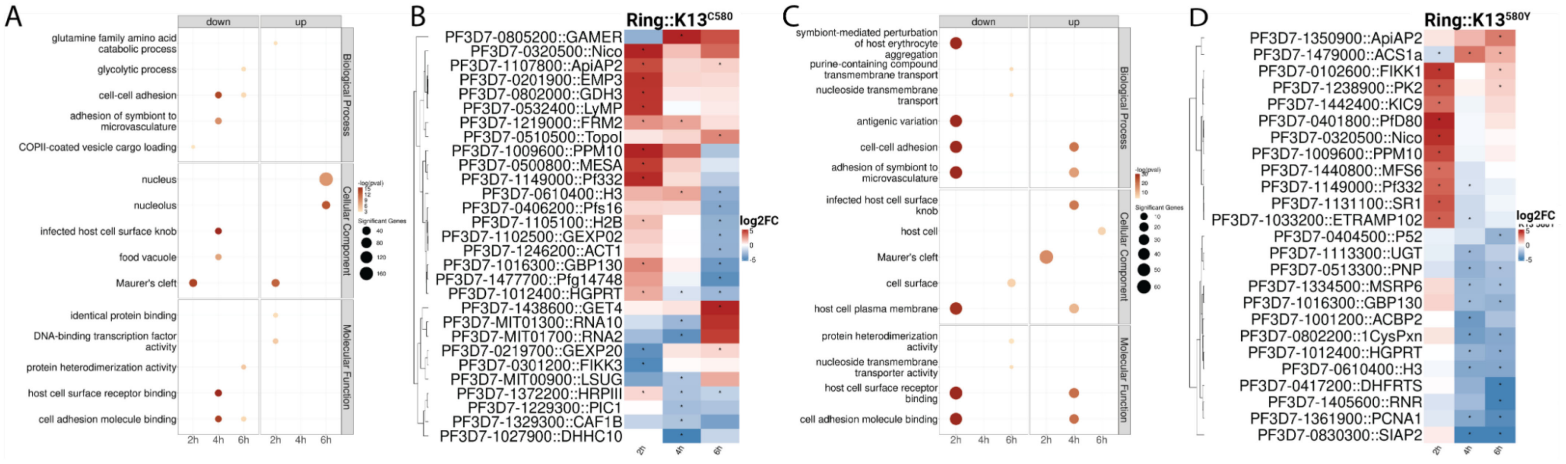
DHA-induced upregulation of exported protein genes, downregulation of glycolysis, and later upregulation of fatty acid metabolism in K13^C580^ and K13^580Y^ rings. Fine-grained DE was performed within each strain. GO analysis was performed over the entire dataset in all differentially expressed genes in the selected VFC subcluster comparisons of K13^C580^ **(A)** or K13^580Y^ **(C)** rings, with the number of significant genes that support that GO Term being the size of the circles (topGO *p*-value < 0.01) and the color being the magnitude of the negative log of the topGO *p*-value. GO terms were filtered to the top 2 significant terms per timepoint and ontology. The union of the top 10 differentially expressed genes (by absolute log2FC) per time point between the VFC subclusters in the highest and lowest DHA likelihoods in K13^C580^ **(B)** or K13^580Y^ **(D)** rings. The log2FC per timepoint is plotted. An asterisk indicates genes that were significantly differentially expressed at an absolute log_2_FC > 1 and a BH-adjusted *p*-value < 0.001. Red boxes are upregulated genes, white boxes indicate genes that were not differentially expressed, and blue boxes are downregulated genes.

Comparing DHA versus DMSO of treatment K13^C580^ rings across timepoints using our fine-grained DE approach (**Figures 2A, B**), the number of differentially upregulated genes increased as treatment continued across the timepoints (2h: 197 genes, 4h: 181 genes, 6h: 345 genes). GO analysis utilizing the significantly differentially expressed genes showed consistent downregulation of glycolytic genes (*ENO* and *LDH*), cell-cell adhesion, food vacuole metabolism, and host cell surface pathways (**Figure 2A**). Upregulated pathways included nuclear, transcriptional factor, and protein-binding pathways. There was upregulation of genes at 2h that are exported proteins in the host cell membrane (*EMP3, MESA*, and *Pf332*). An initial upregulation and then subsequent significant downregulation of histone H3/H2B, exported protein genes (*GEXP02* and *GBP130*), and nucleotide salvage gene *HGPRT* (Keough et al., 1999). Fatty acid metabolic genes *ACS10*, *K13*, and *KIC5* were significantly upregulated at 4h and 6h, with *KIC1* upregulated only at 6h (**Supplementary Table S2**).

Whereas in DHA versus DMSO-treated K13^580Y^ rings across timepoints and after using our fine-grained DE approach (**Figures 2C, D**), the greatest number of DE genes was at 2h, which then decreased over time (2h: 427 genes, 4h: 250 genes, 6h: 218 genes). At 2h, there was an initial upregulation of erythrocyte cell surface genes (*ETRAMP102*, *Pf332, PfD80*, and *FIKK1*). At 4h and 6h post-treatment, there was an upregulation of acyl-CoA synthetase genes involved in fatty acid utilization (*ACS1a*) and downregulation of genes involved in nuclear pathways (*H3*, *HGPRT*, and *PCNA1*). *K13* and *KICs* were significantly upregulated: *KIC9* at 2h, *KIC4* at 4h, and *K13, KIC5*, and *KIC6* at 4h and 6h (**Supplementary Table S2**).

### Fine grained differential expression comparing DHA exposure in Trophozoite-staged K13^C580^ or K13^580Y^

**Figure 3:**
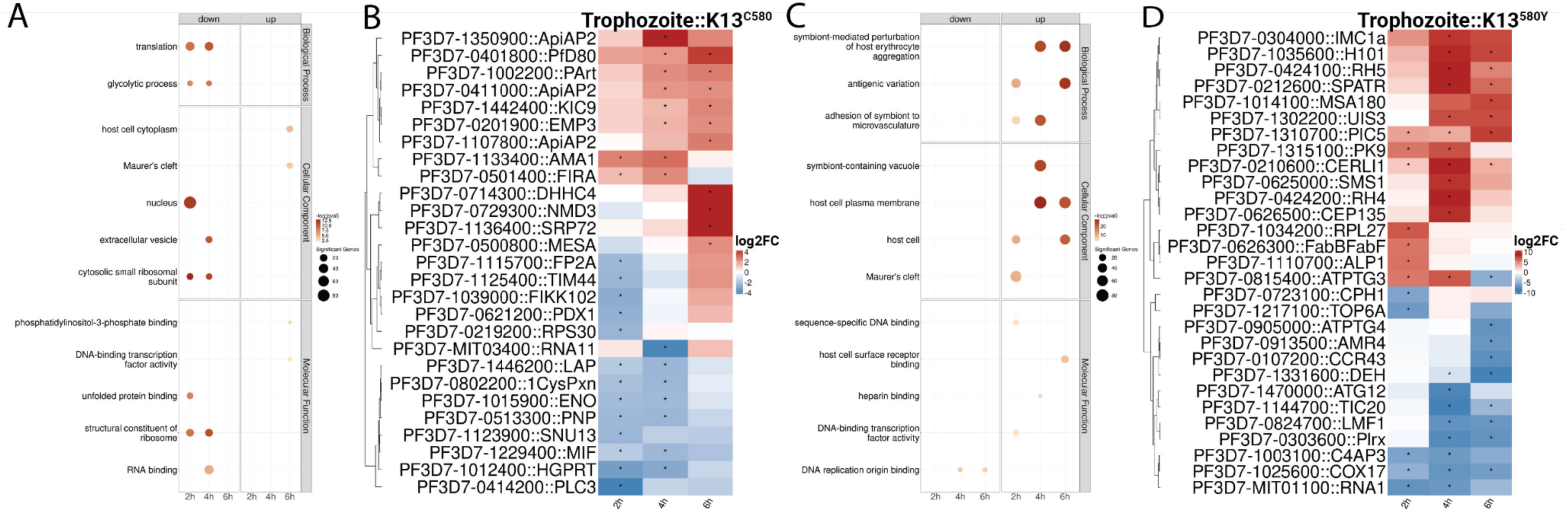
DHA treatment induced protein export genes in K13^C580^ and K13^580Y^ trophozoites, with broad downregulation of mitochondrial and apicoplast pathways in K13^580Y^ trophozoites. Fine-grained DE was performed within each strain. GO analysis was performed over all differentially expressed genes in the selected VFC subcluster comparisons of K13^C580^ **(A)** or K13^580Y^ **(C)** trophozoites, with the number of significant genes that support that GO Term being the size of the circles (topGO *p*-value < 0.01) and the color being the magnitude of the negative log of the topGO *p*-value. GO terms were filtered to the top 2 significant terms per timepoint. The union of the top 10 differentially expressed genes (by absolute log_2_FC) per time point between the VFC subclusters in the highest and lowest DHA likelihoods in K13^C580^ **(B)** or K13^580Y^ **(D)** trophozoites. An asterisk indicates genes that were significantly differentially expressed at an absolute log_2_FC > 1 and a BH-adjusted *p*-value < 0.001. Red boxes are upregulated genes, white boxes indicate genes that were not differentially expressed, and blue boxes are downregulated genes.

Comparing DHA or DMSO treatment in K13^C580^ trophozoites over time using our fine-grained DE approach (**Figures 3A, B**), the number of DE genes decreased over time (2h: 200 genes, 4h: 117 genes, 6h: 44 genes). Translation, nuclear, glycolytic, and ribosomal pathways were downregulated. Artesunate-inducible gene *part* (Natalang et al., 2008; Deplaine et al., 2011) and genes involved with protein export (*PfD80, EMP3, AMA1, FIRA*) were upregulated. Downregulated genes included purine salvage gene *HGPRT*, glycolytic gene *ENO*, protease *LAP*, and peroxidase *1-cys peroxiredoxin*. *KIC1* (at 2h and 4h) and *KIC9* (at 4h and 6h) were significantly upregulated, while *KIC10* was significantly upregulated at 2h (**Supplementary Table S2**).

After DHA treatment in K13^580Y^ trophozoites over time (**Figures 3C, D**), there was greater induction of transcription compared to the DHA-treated K13^C580^ trophozoites, shown after using our fine-grained DE approach (2h: 571 genes, 4h: 1076 genes, 6h: 1249 genes). Across time, there was a strong and broad upregulation of genes related to parasite-erythrocyte interaction and invasion and adhesion to microvasculature, including *SPATR, reticulocyte-binding protein homologue 4 (RH4)/RH5* (Baum et al., 2009)*, merozoite surface protein* (*H101*), and *cytosolically exposed rhoptry leaflet interacting protein 2* (*CERLI*) (Liffner et al., 2020). Mitochondrial and apicoplast pathways were downregulated, containing genes such as *C4AP3, COX17*, and *AMR4* (Meister et al., 2020). *K13* was significantly upregulated at 2h and 4h. *KIC1, KIC5, KIC6, KIC7, KIC4*, and *KIC9* was significantly upregulated across timepoints, *KIC2* was significantly upregulated at 4h and 6h, and *KIC10* was significantly downregulated at 6h (**Supplementary Table S2**).

### Anti-GARP kills K13^C580^ *and* K13^580Y^ *in vitro*

**Figure 4:**
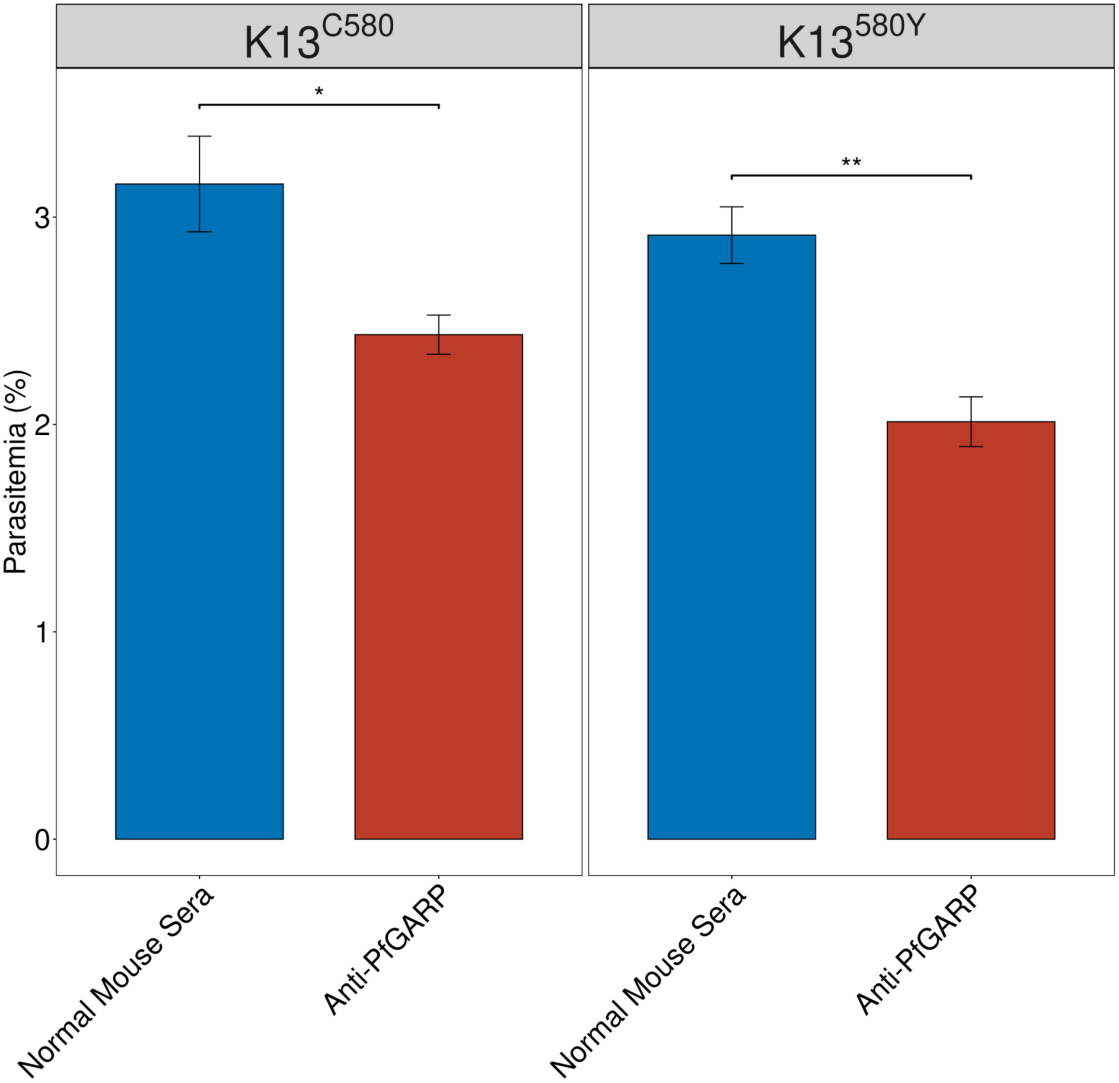
Anti-PfGARP inhibits parasite growth in both artemisinin-sensitive and resistant parasites. Artemisinin-sensitive (K13^C580^) and resistant (K13^580Y^) parasites were synchronized to the ring stage, adjusted to 0.4% parasitemia and 2% hematocrit, and incubated with anti-PfGARP (10% v/v) or normal mouse sera (10% v/v). Parasites were cultured for 72h, followed by quantification of parasitemia by flow cytometry. Bars represent the mean of three technical replicates, error bars represent mean ± standard deviation and significance between treatment groups was assessed with an unpaired t-test with bonferroni adjustment (*p < 0.05, **p < 0.01).

Given GARP expression was particularly elevated in untreated K13^580Y^ compared to K13^C580^ rings (**Figure 1B**), we evaluated if this elevated expression was a result of a functional or aberrant accumulation of the protein in the membrane. We performed growth-inhibition assays (GIAs) using polyclonal mouse anti-GARP antiserum (Raj et al., 2020). We found statistically significant growth inhibition of both K13^C580^ and K13^580Y^ lines after exposure to anti-GARP (**Figure 4**), consistent with the functionality of the pathway in both lines and the upregulation of GARP in the K13^580Y^ line (**Figure 4**). This result suggests that other elevated surface proteins, like GARP, could remain functionally intact and may contribute to the DHA response.

## Discussion

To date, studies of *Plasmodium falciparum* transcriptional responses to artemisinin have largely relied on bulk RNA sequencing of synchronized parasites, limiting resolution across developmental stages and obscuring cell-to-cell heterogeneity (Dondorp et al., 2009; Mok et al., 2015; Zhu et al., 2018; Mok et al., 2021; Zhu et al., 2022). Here, we leveraged single-cell RNA sequencing to comprehensively profile the transcriptional landscape of artemisinin-sensitive (K13^C580^) and artemisinin-resistant (K13^580Y^) isogenic parasites following DHA exposure. This approach revealed stage-specific insights into drug response between these two isogenic lines. As differential expression was performed within individual stages, these results represent stage-resolved transcriptional states associated with DHA exposure. In comparing K13^580Y^ versus K13^C580^ untreated rings, there was an upregulation of exported protein-related genes (notably *GARP, MESA*, and *Pf332*) and genes in metabolic pathways, with upregulated genes enriched in host cell modification and virulence functions (**Figure 1**). Differential expression in trophozoites showed downregulation of mitochondrial and apicoplast genes when comparing K13^580Y^ versus K13^C580^. Together, these expression patterns may indicate that K13^580Y^ parasites have adapted a transcriptional program to increase exported protein pathways while reducing mitochondrial sources of reactive oxygen species to survive drug treatment at the tradeoff of fitness (Zhu et al., 2022; Behrens et al., 2024).

Using MELD, we quantified the extent of experimental perturbation across the lifecycle. DHA likelihood was higher in DHA-treated cells and showed greater separation of treated or control conditions over time (**Supplementary Figure S19**). This finding could be due to the sustained treatment of these samples over the 6h timeframe, resulting in the greater separation of each treatment group over time. We then separated out populations of rings and trophozoites that were most and least perturbed by DHA treatment to perform DE analysis. In K13^C580^ and K13^580Y^ ring stages, we observed upregulation of genes involved in protein export and fatty acid utilization (**Figure 2A**; **Supplementary Table S2**). Fatty acid metabolism is hypothesized to be an alternative energy source to sustain parasite viability upon DHA treatment (Chen et al., 2014; Pires et al., 2023). Pires et al. proposed a stress response upon DHA treatment that consists of the “exportome” of secreted parasite proteins (Hiller et al., 2004), potentially acting in synergy to facilitate lipid transport for fatty acid metabolism (Pires et al., 2023). In our data, both lines showed downregulation of glycolytic processes and upregulation of exported protein and fatty acid metabolic genes (**Figure 2**). DHA-treated ring-staged parasites’ broad activation of exported proteins and increase in fatty acid metabolic pathway genes suggest a shift to fatty acid metabolism to respond to DHA-induced stress. Future studies aiming to inhibit acyl-CoA synthetases, such as ACS10, may be a promising avenue to bolstering DHA treatment (Bopp et al., 2023).

In both K13^C580^ and K13^580Y^ DHA-treated trophozoites, there was an upregulation of genes coding for exported proteins and invasion mediators. Additionally, there was a downregulation of metabolism and nuclear pathways in both strains (**Figure 3**). These results support previous reports of translational down-regulation after DHA treatment (Mok et al., 2021). Interestingly, both DHA-treated K13^C580^ and K13^580Y^ trophozoites showed downregulation of genes involved in glycolysis, purine metabolism, and fatty acid metabolism (**Figure 3**; **Supplementary Table S2**). Whereas only in DHA-treated K13^580Y^ trophozoites, we saw broad downregulation of mitochondrial and apicoplast pathway gene expression. This result suggests that the K13 mutation may alter different metabolic pathways in response to DHA treatment, similar to prior work showing differential mitochondrial pathway use in sensitive versus resistant lines (Mok et al., 2021). The transcriptional metabolic changes after DHA treatment suggest a metabolic shift in energy utilization and organellar activity, but this finding requires validation through metabolomic profiling and targeted genetic or pharmacologic inhibition of key metabolic regulators. Additionally, the perturbation response was limited by the distribution of stages present at the time of DHA treatment, so robust DHA response phenotypes over more replicates and in sexually committed rings, gametocytes, and schizonts is an important avenue for further investigation.

Throughout our analysis, we saw differential upregulation or downregulation of transcriptional regulators, such as *ApiAP2* family genes (**Figures 2**, **3**). Recently, Yu et al. identified epigenetic modulators that contribute to ring stage survival after DHA treatment, notably the histone acetyltransferase *MYST* (Yu et al., 2025). Our findings showing altered expression of transcription factor activity underscore the importance of further investigating transcriptional regulation of the artemisinin response.

The K13 protein exhibits sequence similarity to a class of Kelch/BTB/POZ ubiquitination adaptors and functions within the endocytosis pathway required for hemoglobin uptake from the host cell (Ariey et al., 2014; Xie et al., 2020), which is critical for the activation of the endoperoxide bridge of ART (Meshnick, 2002). *K13* mutations in the propeller domain are thought to partially reduce the normal function of the K13 protein, which in turn is thought to diminish hemoglobin endocytosis and ART activation (Yang et al., 2019; Birnbaum et al., 2020). Reduced hemoglobin uptake limits the availability of Fe^2+^ heme to activate ART and kill ring-stage parasites via alkylation of proximal proteins, leading to reduced ART killing (Meshnick, 2002; Tilley et al., 2016). At baseline, *K13* was downregulated in K13^580Y^ ring-staged parasites compared to K13^C580.^ K13’s function in facilitating hemoglobin entry has been shown to be aided by a complex of other proteins, known as the Kelch13 interacting candidates (KICs) (Birnbaum et al., 2020). Downregulation of hemoglobin peptides upon DHA treatment occurs regardless of *K13* genotype (Mok et al., 2021). There was a broad upregulation of *KIC* genes that are localized to the K13 endocytosis compartment (such as *KIC4, KIC5*, and *KIC7*) (Schmidt et al., 2023) across rings and trophozoites of both lines after DHA treatment, possibly as a compensatory measure to preserve hemoglobin endocytosis.

Differential expression in ring-staged K13^580Y^ versus K13^C580^ showed upregulated levels of GARP in K13^580Y^ (**Figure 1C**). GARP was recently identified as a therapeutic target (Raj et al., 2020), while its function remains to be characterized. We observed elevated expression of GARP in K13^580Y^ compared to K13^C580^ before treatment, possibly as a result of a compensatory increase in the expression of the “exportome” (Maier et al., 2008). Given gene expression changes can correlate with function or represent dysregulation (e.g., increased turnover), we examined whether GARP still retained function as a therapeutic target, finding statistically significant anti-GARP killing of both K13^C580^ and K13^580Y^ parasites (**Figure 4**). This suggests that GARP upregulation could be a consequence of a coordinated “exportome” response across genetic backgrounds, but further mechanistic investigations are required.

Our study provides a view of the single-cell transcriptional landscape underlying artemisinin response in *Plasmodium falciparum*, revealing both shared stress pathways and resistance-associated adaptations. Together, these findings not only advance our understanding of the molecular basis of ART-R but also provide a framework for rational design of next-generation therapies that exploit vulnerabilities in resistant parasites. By leveraging single-cell RNA sequencing, we demonstrate that DHA-treated parasites may deploy a distinct transcriptional program consistent with enhanced protein export and metabolic flexibility, including fatty acid utilization, while downregulating organellar functions. Importantly, the inhibition of GARP and its possible role in sustaining parasite growth through lipid metabolism underscores the potential of targeting exportome components to overcome resistance. Future work integrating transcriptomic, proteomic, and functional studies will be critical to better assess and translate these insights into effective interventions against evolving malaria resistance.

## Data Availability

All sequence data used in this study can be found in the NCBI Sequence Read Archive (SRA) (accession PRJNA1049964). Raw data matrices and code are available at https://zenodo.org/records/20344254.
