# Supplementary material for: Single-cell transcriptional changes of artemisinin-sensitive K13C580 and artemisinin-resistant K13580Y Plasmodium falciparum upon dihydroartemisinin exposure
Source: Front Cell Infect Microbiol. 2026 Jul 28;16:1816105. doi: 10.3389/fcimb.2026.1816105 (PMC13457298; doi:10.3389/fcimb.2026.1816105)
Supplement: Supplementary file 1 [file DataSheet1.docx]

**Supplemental Materials for**

**Single-cell transcriptional changes of artemisinin-sensitive K13^C580^ and artemisinin-resistant K13^580Y^ *Plasmodium falciparum* upon dihydroartemisinin exposure**

Sean V. Connelly^1^, Clark Cunningham^1^, Ian Petrow^2,3^, Nazrin Rustamzade^2^, Jenna Zuromski^1^, Deborah M. Sipolski^2^, Christian P. Nixon^2^, Jonathan D. Kurtis^2,3^, Jonathan J. Juliano^1,4,5,6^**^+^**, Jeffrey A. Bailey^2^, Cliff I. Oduor^2*^

1. Division of Infectious Diseases, Department of Medicine, University of North Carolina, Chapel Hill, NC, USA

2. Department of Pathology and Laboratory Medicine, Brown University, Providence, RI, USA

3. Center for International Health Research, Providence, RI, USA

4. Curriculum in Genetics and Molecular Biology, School of Medicine, University of North Carolina, Chapel Hill, NC, USA

5. Department of Epidemiology, Gillings School of Global Public Health, University of North Carolina, Chapel Hill, NC, USA

6. Institute for Global Health and Infectious Diseases, University of North Carolina, Chapel Hill, NC, USA

Supplemental Methods……………………………………………………………………………………………	3

Supplemental References………………………………………………………………………………………..	5

Supplemental Figure S1……………………………………………………………………………………………	7

Supplemental Figure S2……………………………………………………………………………………………	8

Supplemental Figure S3……………………………………………………………………………………………	9

Supplemental Figure S4……………………………………………………………………………………………	10

Supplemental Figure S5……………………………………………………………………………………………	11

Supplemental Figure S6……………………………………………………………………………………………	12

Supplemental Figure S7……………………………………………………………………………………………	13

Supplemental Figure S8……………………………………………………………………………………………	14

Supplemental Figure S9……………………………………………………………………………………………	15

Supplemental Figure S10…………………………………………………………………………………………..16

Supplemental Figure S11…………………………………………………………………………………………..17

Supplemental Figure S12…………………………………………………………………………………………..18

Supplemental Figure S13…………………………………………………………………………………………..19

Supplemental Figure S14…………………………………………………………………………………………..20

Supplemental Figure S15…………………………………………………………………………………………..21

Supplemental Figure S16…………………………………………………………………………………………..22

Supplemental Figure S17…………………………………………………………………………………………..23

Supplemental Figure S18…………………………………………………………………………………………..24

Supplemental Figure S19…………………………………………………………………………………………..25

Supplemental Figure S20…………………………………………………………………………………………..26

Supplemental Figure S21…………………………………………………………………………………………..27

Supplemental Figure S22…………………………………………………………………………………………..28

Supplemental Figure S23…………………………………………………………………………………………..29

Supplemental Figure S24…………………………………………………………………………………………..30

Supplemental Figure S25…………………………………………………………………………………………..31

Supplemental Figure S26…………………………………………………………………………………………..32

Supplemental Figure S27…………………………………………………………………………………………..33

Supplemental Table 1………….……………………………………………………………………………………34

Supplemental Table 2……………………………………………………………………………………………….35

Supplemental Table 3……………………………………………………………………………………………….36

Supplemental Table 4……………………………………………………………………………………………….37

Supplemental Table 5……………………………………………………………………………………………….38

**Supplemental Methods**

**SeqWell Library Preparation and Sequencing.** Briefly, 10-15,000 *Pf* infected RBCs from each time point and treatment condition were loaded onto a functionalized polydimethylsiloxane (PDMS) array preloaded with uniquely barcoded mRNA capture beads (Chemgenes; Macosko-2011-10(V+), Wilmington, MA). After cells had settled into wells, the arrays were then sealed with a hydroxylated polycarbonate membrane with a pore size of 10 nm, facilitating buffer exchange while confining biological molecules (such as DNA and RNA) within each well. Following membrane-sealing, subsequent buffer exchange permitted cell lysis, mRNA transcript hybridization to beads, and bead removal before proceeding with reverse transcription. The obtained bead-bound cDNA product then underwent Exonuclease I treatment to remove excess primer before proceeding with second-strand synthesis and PCR amplification of the cDNA product. The cDNA products were purified using SPRIselect beads (Beckman Coulter, Brea, CA), and were run on a Fragment Analyzer (Agilent Technologies, Santa Clara, CA) to verify the cDNA fragment sizes to be between 0.7-2kbps. The cDNA libraries were then fragmented and amplified for sequencing using the Illumina Nextera XT DNA Library Preparation kit (Illumina, FC-131-1096, San Diego, CA) using custom primers that enabled the specific amplification of only the 3’ ends. The libraries were purified, quantified, and then sequenced on a NextSeq550 system using high output 75 cycle kits (Illumina) at an average of 20,000 reads per cell.

**MIP capture, sequencing, and variant calling.** DNA isolates for the isogenic cell lines were captured and sequenced using a drug-resistance MIP panel, which is designed to target mutations and genes associated with antimalarial resistance [(1,2)](https://paperpile.com/c/HvL74X/K8x6x+kDghC). MIP capture and library preparation were performed as previously described [(3)](https://paperpile.com/c/HvL74X/MRXO3). Sequencing was conducted using an Illumina NextSeq 550 instrument (150 bp paired-end reads) at Brown University’s genomics core (Rhode Island, USA). The MIPtools (v.0.19.12.13; https://github.com/bailey-lab/MIPTools) bioinformatic pipeline was used for processing of sequencing data and variant calling as previously described[(1,3,4)](https://paperpile.com/c/HvL74X/K8x6x+WFv2D+MRXO3). The drug-resistance panel included known SNPs in *pfcrt, pfdhfr, pfdhps, pfmdr1, K13,* and other putative drug-resistance genes and has been described elsewhere [(1)](https://paperpile.com/c/HvL74X/K8x6x). MIP genotyping results are contained in **Supplemental Table 5**.

**Cell Annotation Pipeline.** Each dataset was processed as follows: the *cell x gene* expression matrix was normalized with SCTransform [(5,6)](https://paperpile.com/c/HvL74X/PPBFK+6BzxG) before dimensionality reduction through uniform manifold approximation (UMAP) [(7)](https://paperpile.com/c/HvL74X/b9zih) and clustering through the Leiden algorithm [(8)](https://paperpile.com/c/HvL74X/bQByd). After unsupervised clustering, a first pass was run on our stage annotation pipeline. We calculated pseudobulk gene expression of each cluster, visualizing both literature guided stage markers [(9–11)](https://paperpile.com/c/HvL74X/Z74Zg+7BpYF+1KzZK) and the top 5 differentially expressed genes per cluster. We correlated our pseudobulk clusters to two bulk RNA sequencing datasets, one a time course experiment measuring RNA transcriptional abundance over the asexual lifecycle [(12)](https://paperpile.com/c/HvL74X/hxTsi) and the other focusing on merozoite stages of *P. falciparum* [*(9)*](https://paperpile.com/c/HvL74X/Z74Zg). Next, we mapped our scRNAseq cells to the most recent malaria cell atlas of *P. falciparum* [*(10)*](https://paperpile.com/c/HvL74X/7BpYF) and to a dataset of SmartSeq2 plate based *P. berghei* scRNAseq subsetted to one-to-one orthologs [(11)](https://paperpile.com/c/HvL74X/1KzZK). Doublets detected by scrublet and doubletFinder were filtered (**Supplemental Figure S7** and **S8**). Any cells correlated to merozoites were filtered because there were very few merozoites present in only 2/14 datasets. The remaining cells of all datasets were integrated with Harmony [(13)](https://paperpile.com/c/HvL74X/Z7zHg). The stage annotation pipeline was run again, where any cells that were not within the asexual or sexual developmental trajectories were filtered out as doublets (**Supplemental Figure S9** and **S10**). After those doublets were filtered, the stage annotation pipeline was run for a final time to result in the final stage annotations (**Supplemental Figure S11**-**S13**). The proportions of each stage are shown in **Supplemental Figure S14**. Plots generated after each step in this pipeline are presented in an interactive HTML at https://zenodo.org/records/20344254/K13_mt_v_wt_sc_v1/clustering_summary.html.

**Differential Expression (DE) Pipeline (Supplemental Figure S16).**

- Differential Expression Workflow:
  - In **Figure 1**, differential expression was performed between K13^580Y^ vs. K13^C580^ stages at 0h utilizing MAST [(14)](https://paperpile.com/c/HvL74X/adFa). Statistical thresholds at a BH-adjusted p value < 0.001 and an absolute log2FC > 1 were implemented in all DE analyses with MAST.
- Fine Grained DE Workflow:
  - Optimizing MELD: In **Figures 2 and 3**, we perform a fine grained differential expression (DE) workflow utilizing MELD. We filtered the dataset to only DMSO or DHA treated cells for fine grained DE. After separating each strain, the normalized cell x gene expression matrix was exported from Seurat and imported as an annotated data object [(15)](https://paperpile.com/c/HvL74X/I1Euw) in Python (v3.9.15). PCA was performed on each gene expression matrix. The top 100 PCs from the PCA embedding were used to create a Potential of Heat diffusion for Affinity-based Transition Embedding (PHATE), with default settings [(16)](https://paperpile.com/c/HvL74X/YR92H). Parameter optimization for MELD was performed on the normalized cell x gene expression matrix over a range of k-nearest neighbors (KNNs) and beta, a smoothing parameter, values. During parameter optimization, a random probability density function (PDF) is generated over the data, and cells are randomly assigned to DHA or DMSO treatment conditions. MELD was then fit to the normalized cell x gene matrix with the given parameters, and the Mean Square Error (MSE) between MELD’s DHA likelihoods and the ground truth PDF was calculated (see **Supplemental Figure S17-18**). This process was repeated 25 times for each KNN, over the range of beta values. The average MSE was calculated, and the parameters with the lowest mean MSE (shown as a red dot in **Supplemental Figure S18**) were selected. The optimal parameters for each strain were then used downstream for running MELD.
  - Running MELD: For each strain, MELD was run per time point, with the optimal parameters per strain, the timepoint’s PCA embedding, and treatment condition (DMSO or DHA) labels as input, similar to previous work [(17)](https://paperpile.com/c/HvL74X/6CMIb). The output was a continuous score of experimental perturbation, referred to as the DHA likelihood.
  - Vertex Frequency Clustering (VFC): For each malaria cell cycle stage, the PCA embedding, treatment labels, and DHA likelihoods were used as input for vertex frequency clustering (VFC). This PCA embedding was then used by PHATE to visualize the VFC results. VFC results are in **Supplemental Figures S20-S24**.
  - MAST DE: Differential expression between the VFC clusters with the highest DHA likelihood per timepoint was performed with the MAST [(14)](https://paperpile.com/c/HvL74X/adFa) package. Statistical thresholds for the BH-adjusted and absolute log2FC were implemented as above. All differential expression results are present in **Supplemental Table S2**. For heatmap visualization in **Figures 1-3**, hypervariable genes were removed due to their overwhelming signal in the dataset. Then, annotated genes were selected and ranked by log2FC to find the top 30 genes by absolute log2FC for **Figure 1** or top 10 genes by absolute log2FC per timepoint for **Figures 2-3**. The vectors of the top 10 (**Figures 2**-**3**) genes per timepoint were concatenated and filtered to unique genes.

**Supplemental References**

1.	[Aydemir O, Janko M, Hathaway NJ, Verity R, Mwandagalirwa MK, Tshefu AK, et al. Drug-resistance and population structure of Plasmodium falciparum across the Democratic Republic of Congo using high-throughput molecular inversion probes. J Infect Dis. 2018 Aug 14;218(6):946–55.](http://paperpile.com/b/HvL74X/K8x6x)

2.	[Verity R, Aydemir O, Brazeau NF, Watson OJ, Hathaway NJ, Mwandagalirwa MK, et al. The impact of antimalarial resistance on the genetic structure of Plasmodium falciparum in the DRC. Nat Commun. 2020 Apr 30;11(1):2107.](http://paperpile.com/b/HvL74X/kDghC)

3.	[Moser KA, Madebe RA, Aydemir O, Chiduo MG, Mandara CI, Rumisha SF, et al. Describing the current status of Plasmodium falciparum population structure and drug resistance within mainland Tanzania using molecular inversion probes. Mol Ecol. 2021 Jan;30(1):100–13.](http://paperpile.com/b/HvL74X/MRXO3)

4.	[Fola AA, Feleke SM, Mohammed H, Brhane BG, Hennelly CM, Assefa A, et al. Plasmodium falciparum resistant to artemisinin and diagnostics have emerged in Ethiopia. Nat Microbiol. 2023 Oct;8(10):1911–9.](http://paperpile.com/b/HvL74X/WFv2D)

5.	[Hafemeister C, Satija R. Normalization and variance stabilization of single-cell RNA-seq data using regularized negative binomial regression. Genome Biol. 2019 Dec 23;20(1):296.](http://paperpile.com/b/HvL74X/PPBFK)

6.	[Choudhary S, Satija R. Comparison and evaluation of statistical error models for scRNA-seq. Genome Biol. 2022 Jan 18;23(1):27.](http://paperpile.com/b/HvL74X/6BzxG)

7.	[Becht E, McInnes L, Healy J, Dutertre CA, Kwok IWH, Ng LG, et al. Dimensionality reduction for visualizing single-cell data using UMAP. Nat Biotechnol [Internet]. 2018 Dec 3; Available from:](http://paperpile.com/b/HvL74X/b9zih) <http://dx.doi.org/10.1038/nbt.4314>

8.	[Traag VA, Waltman L, van Eck NJ. From Louvain to Leiden: guaranteeing well-connected communities. Sci Rep. 2019 Mar 26;9(1):5233.](http://paperpile.com/b/HvL74X/bQByd)

9.	[Reers AB, Bautista R, McLellan J, Morales B, Garza R, Bol S, et al. Histone modification analysis reveals common regulators of gene expression in liver and blood stage merozoites of Plasmodium parasites. Epigenetics Chromatin. 2023 Jun 15;16(1):25.](http://paperpile.com/b/HvL74X/Z74Zg)

10.	[Dogga SK, Rop JC, Cudini J, Farr E, Dara A, Ouologuem D, et al. A single cell atlas of sexual development in Plasmodium falciparum. Science. 2024 May 3;384(6695):eadj4088.](http://paperpile.com/b/HvL74X/7BpYF)

11.	[Howick VM, Russell AJC, Andrews T, Heaton H, Reid AJ, Natarajan K, et al. The Malaria Cell Atlas: Single parasite transcriptomes across the complete Plasmodium life cycle. Science [Internet]. 2019 Aug 23;365(6455). Available from:](http://paperpile.com/b/HvL74X/1KzZK) <http://dx.doi.org/10.1126/science.aaw2619>

12.	[Painter HJ, Chung NC, Sebastian A, Albert I, Storey JD, Llinás M. Genome-wide real-time in vivo transcriptional dynamics during Plasmodium falciparum blood-stage development. Nat Commun [Internet]. 2018 Jul 9;9(1). Available from:](http://paperpile.com/b/HvL74X/hxTsi) <http://dx.doi.org/10.1038/s41467-018-04966-3>

13.	[Korsunsky I, Millard N, Fan J, Slowikowski K, Zhang F, Wei K, et al. Fast, sensitive and accurate integration of single-cell data with Harmony. Nat Methods. 2019 Dec;16(12):1289–96.](http://paperpile.com/b/HvL74X/Z7zHg)

14.	[Finak G, McDavid A, Yajima M, Deng J, Gersuk V, Shalek AK, et al. MAST: a flexible statistical framework for assessing transcriptional changes and characterizing heterogeneity in single-cell RNA sequencing data. Genome Biol. 2015 Dec 10;16:278.](http://paperpile.com/b/HvL74X/adFa)

15.	[Wolf FA, Angerer P, Theis FJ. SCANPY: large-scale single-cell gene expression data analysis. Genome Biol. 2018 Feb 6;19(1):15.](http://paperpile.com/b/HvL74X/I1Euw)

16.	[Moon KR, van Dijk D, Wang Z, Gigante S, Burkhardt DB, Chen WS, et al. Visualizing structure and transitions in high-dimensional biological data. Nat Biotechnol. 2019 Dec;37(12):1482–92.](http://paperpile.com/b/HvL74X/YR92H)

17.	[Burkhardt DB, Stanley JS 3rd, Tong A, Perdigoto AL, Gigante SA, Herold KC, et al. Quantifying the effect of experimental perturbations at single-cell resolution. Nat Biotechnol. 2021 May;39(5):619–29.](http://paperpile.com/b/HvL74X/6CMIb)

18.	[Wolock SL, Lopez R, Klein AM. Scrublet: Computational identification of cell Doublets in Single-cell transcriptomic data. Cell Syst. 2019 Apr 24;8(4):281–91.e9.](http://paperpile.com/b/HvL74X/njqVA)

19.	[McGinnis CS, Murrow LM, Gartner ZJ. DoubletFinder: Doublet detection in single-cell RNA sequencing data using artificial nearest neighbors. Cell Syst. 2019 Apr 24;8(4):329–37.e4.](http://paperpile.com/b/HvL74X/Garv6)

20.	[Neavin D, Senabouth A, Arora H, Lee JTH, Ripoll-Cladellas A, sc-eQTLGen Consortium, et al. Demuxafy: improvement in droplet assignment by integrating multiple single-cell demultiplexing and doublet detection methods. Genome Biol. 2024 Apr 15;25(1):94.](http://paperpile.com/b/HvL74X/ABiRP)

21.	[Kiselev VY, Yiu A, Hemberg M. scmap: projection of single-cell RNA-seq data across data sets. Nat Methods. 2018 May;15(5):359–62.](http://paperpile.com/b/HvL74X/vRqdJ)

22.	[Street K, Risso D, Fletcher RB, Das D, Ngai J, Yosef N, et al. Slingshot: cell lineage and pseudotime inference for single-cell transcriptomics. BMC Genomics [Internet]. 2018 Dec;19(1). Available from:](http://paperpile.com/b/HvL74X/0zRIP) <http://dx.doi.org/10.1186/s12864-018-4772-0>

**Supplemental Figure S1 - Read Mapping Percentages to Human and *Plasmodium falciparum* genomes.** A) For each sample, STAR alignment was performed, aligning to a combined human and *P. falciparum* genome. The total read counts input into STAR was shown as the second column, with species specific read counts or percentages in the subsequent columns. B) The last two columns of (A) were plotted as a bar graph for each sample.

**Supplemental Figure S2 - Quality Control Filtering of K13^C580^ cells.** Cells are plotted by the number of UMI counts (nCount_RNA) vs. the number of genes (nFeature_RNA), while being colored by the percent mitochondrial gene expression (percent.mt). Filtering thresholds are shown in red (greater than 500 UMis for nCount_RNA and greater than 300 genes for nFeature_RNA). Additionally, cells with less than 20% mitochondrial gene expression were retained.

**Supplemental Figure S3 - Quality Control Filtering of K13^580Y^ cells.** Cells are plotted by the number of UMI counts (nCount_RNA) vs. the number of genes (nFeature_RNA), while being colored by the percent mitochondrial gene expression (percent.mt). Filtering thresholds are shown in red (greater than 500 UMis for nCount_RNA and greater than 300 genes for nFeature_RNA). Additionally, cells with less than 20% mitochondrial gene expression were retained.

**Supplemental Figure S4 - Quality Control Filtering of Genes in the K13^C580^ Dataset.** Each plot shows a histogram of genes binned by the number of UMI counts over all cells. Genes were retained with a threshold determined by the maximum between two options; if they were expressed in at least 10 cells or expressed in at least 1% of all cells.

**Supplemental Figure S5 - Quality Control Filtering of Genes in the K13^580Y^ Dataset.** Each plot shows a histogram of genes binned by the number of UMI counts over all cells. Genes were retained with a threshold determined by the maximum between two options; if they were expressed in at least 10 cells or expressed in at least 1% of all cells.

**Supplemental Figure S6 - Cell Annotation Pipeline.** A) Malaria stages were annotated through visualization of marker genes per malaria stage, visualizing the top 5 differentially expressed genes in each cluster, performing pseudobulk correlation to both Painter *et al.* [*(12)*](https://paperpile.com/c/HvL74X/hxTsi) and Reers *et al.* [(9)](https://paperpile.com/c/HvL74X/Z74Zg) and mapping to the original [(11)](https://paperpile.com/c/HvL74X/1KzZK) and updated malaria cell atlas datasets [(10)](https://paperpile.com/c/HvL74X/7BpYF). B) Each individual dataset underwent unsupervised clustering and then entered the stage annotation pipeline described in A. Afterwards, any doublets detected by scrublet [(18)](https://paperpile.com/c/HvL74X/njqVA) or doubletFinder [(19)](https://paperpile.com/c/HvL74X/Garv6) were filtered. Lastly, any merozoites were filtered out of the dataset, as they were very few merozoites across the datasets. C) After individual dataset filtering, datasets were integrated with Harmony [(13)](https://paperpile.com/c/HvL74X/Z7zHg) and underwent stage annotation. Any cells that were not on the developmental trajectory were labeled as doublets and subsequently filtered. Filtered datasets were reintegrated and annotated for a final time. Plots of the individual and merged datasets are all encompassed in https://zenodo.org/records/20344254/K13_mt_v_wt_sc_v1/clustering_summary.html. Created in BioRender. Connelly, S. (2026) https://BioRender.com/pqvp4ys.

**Supplemental Figure S7** **- Doublets Detected in the K13^C580^ Dataset.** The number of doublets was estimated to be 2.37% based on previous SeqWell publications. This value was input into both scrublet and doubletFinder to simulate doublets. We utilized 0.13 as the doublet detection threshold in scrublet and the implementation of doubletFinder in the Demuxafy [(20)](https://paperpile.com/c/HvL74X/ABiRP) package to simulate doublets.

**Supplemental Figure S8 - Doublets Detected in the K13^580Y^ Dataset.** The number of doublets was estimated to be 2.37% based on previous SeqWell publications. This value was input into both scrublet and doubletFinder to simulate doublets. We utilized 0.13 as the doublet detection threshold in scrublet and the implementation of doubletFinder in the Demuxafy [(20)](https://paperpile.com/c/HvL74X/ABiRP) package to simulate doublets.

**Supplemental Figure S9 - Doublets Detected after Integrating in K13^C580^.** After integration of the K13^C580^ and K13^580Y^ datasets, doublets were labeled as those cells embedded outside of the asexual and sexual lifecycle trajectories.

**Supplemental Figure S10 - Doublets Detected after Integrating in K13^580Y^.** After integration of the K13^C580^ and K13^580Y^ datasets, doublets were labeled as those cells embedded outside of the asexual and sexual lifecycle trajectories.

**Supplemental Figure S11 - Unsupervised Clustering, Mapping to *Dogga et al.*, and correlation to Painter *et al.*.** A) After filtering out all doublets and merozoites, unsupervised clustering was performed over the entire integrated dataset. B) Each cell was correlated to a malaria single cell RNA sequencing atlas consisting of asexual and sexual stages using scmap [(10,21)](https://paperpile.com/c/HvL74X/7BpYF+vRqdJ). (C) The correlation results between the clusters annotated as asexual stages and the Painter *et al.* dataset. Other clusters were annotated based on marker genes (cluster 5 as sexually committed rings (**Supplemental Figure S12**) and clusters 7 and 8 as gametocytes (**Supplemental Figure S13**).

**Supplemental Figure S12 - Expression of Sexually Committed Ring Markers Over Integrated Dataset.** Expression of sexually committed ring markers (*AP2-G*, *Surfin 1.2* and *Surfin 13.1*) from Prajapati *et al.* 2020 are shown over the integrated dataset’s UMAP. The expression is concentration in Cluster 5.

**Supplemental Figure S13 - Expression of Gametocyte Markers Over Integrated Dataset.** Expression of gametocyte markers from Dogga *et al.* are plotted over the entire integrated dataset. Clusters 7 and 8 show the highest expression of these markers.

**Supplemental Figure S14 - Stage Distribution Per Strain Over Each Timepoint.** For each strain and timepoint, a stacked bar plot is plotted that shows the number of each parasite stage.

**Supplemental Figure S15. Pseudotime over the asexual cycle, separated by strain.** Pseudotime was calculated using slingshot for each strain (top: K13^C580^, bottom: K13^580Y^) over the ring, trophozoite, and schizont stages. Each cell is assigned a pseudotime value. Lower values correspond to earlier developmental pseudotime (ring stage) and are colored purple. Pseudotime values then increase through the trophozoite stage (green) and the schizont stage (yellow).

**Supplemental Figure S16. Schematic of Differential Expression Workflow.** **A) Baseline Differential Expression**. The differential expression workflow is shown here. In **Figure 1**, K13^580Y^ vs. K13^C580^ parasites were compared per stage and per timepoint using MAST to obtain a baseline DE phenotype between these strains. **B) Fine Grained Differential Expression.** The fine grained differential expression workflow is shown here. MELD was fit to each strain’s normalized cell x gene expression matrix, Per timepoint, the strain’s PCA embedding and treatment condition (DMSO or DHA) labels were input into MELD, with these optimized parameters, to estimate the DHA likelihood. For each malaria cell cycle stage, the PCA embedding, treatment labels, and DHA likelihoods were input into VFC. Then, the VFC clusters with the highest vs. lowest DHA likelihoods were compared with MAST per stage and per timepoint [(14)](https://paperpile.com/c/HvL74X/adFa). Created in BioRender. Connelly, S. (2026) https://BioRender.com/fu7k28p.

**Supplemental Figure S17. PHATE Embedding.** Normalized counts exported from the integrated Seurat object were exported and used to create a PHATE embedding for each strain (top: K13^C580^, bottom: K13^580Y^). Cells are colored by stage annotation.

**Supplemental Figure S18. Optimization of MELD algorithm over different KNN (y axis) and beta (x axis) parameters.** As described in the Supplemental Methods, MELD was fit to each strain’s normalized *cell x gene* matrix separately and over a range of KNN and beta values. The red dot on each plot is the optimal parameters used to run MELD for each line.

**Supplemental Figure S19. DHA treated parasites have higher DHA likelihoods over the lifecycle than DMSO treated parasites.** Pseudotime, calculated per strain through slingshot [(22)](https://paperpile.com/c/HvL74X/0zRIP), is plotted on the x-axis while the DHA likelihood output from MELD is plotted on the y-axis for each facet of the figure. Cells are colored by stage annotation (A) or treatment condition (B). The left column plots K13^C580^ cells while the right column plots K13^580Y^ cells. Each row is a different timepoint, with the first row being the 2 hour timepoint, middle row being the 4 hour timepoint and the last row being the 6 hour timepoint.

**Supplemental Figure S20. Vertex frequency clustering (VFC) results for sexually committed rings.** For each timepoint, the sexually committed ring stage was sub-clustered using Vertex Frequency Clustering. The first row (A,C,E) of each timepoint is K13^C580,^ while the second row (B,D,F) is K13^580Y^. For each timepoint, the clusters with the highest DHA likelihood were selected for differential expression.

**Supplemental Figure S21. Vertex frequency clustering (VFC) results for rings.** For each timepoint, the ring stage was sub-clustered using Vertex Frequency Clustering. The first row (A,C,E) of each timepoint is K13^C580,^ while the second row (B,D,F) is K13^580Y^. For each timepoint, the clusters with the highest DHA likelihood were selected for differential expression.

**Supplemental Figure S22. Vertex frequency clustering (VFC) results for trophozoites.** For each timepoint, the trophozoite stage was sub-clustered using Vertex Frequency Clustering. The first row (A,C,E) of each timepoint is K13^C580,^ while the second row (B,D,F) is K13^580Y^. For each timepoint, the clusters with the highest DHA likelihood were selected for differential expression.

**Supplemental Figure S23. Vertex frequency clustering (VFC) results for schizonts.** For each timepoint, the schizont stage was sub-clustered using Vertex Frequency Clustering. The first row (A,C,E) of each timepoint is K13^C580,^ while the second row (B,D,F) is K13^580Y^. For each timepoint, the clusters with the highest DHA likelihood were selected for differential expression.

**Supplemental Figure S24. Vertex frequency clustering (VFC) results for gametocytes.** For each timepoint, the gametocyte stage was sub-clustered using Vertex Frequency Clustering. The first row (A,C,E) of each timepoint is K13^C580,^ while the second row (B,D,F) is K13^580Y^. For each timepoint, the clusters with the highest DHA likelihood were selected for differential expression.

**Supplemental Figure S25. Similar pattern of differentially expressed genes using VFC cluster differential expression compared to stage differential expression at the 2 hour timepoint.** Volcano plots in each panel show the average log2FC on the x axis and the negative log10 of the BH-adjusted p value output from MAST [(14)](https://paperpile.com/c/HvL74X/adFa) on the y axis. Colors denote different levels of significance; non significant genes are in grey, genes with an absolute average log2FC > 1 are green, genes with a BH-adjusted p value < 0.001 are in blue and significant genes with an absolute log2FC > 1 and a BH-adjusted p value < 0.001 are in red. The first row represents differential expression results when comparing between DHA vs. DMSO conditions within a stage for the 2 hour timepoint, between K13^C580^ rings (A), K13^580Y^ rings (B), K13^C580^ trophozoites (C) and K13^580Y^ trophozoites (D). The second row represents differential expression results when comparing the VFC cluster with the highest DHA likelihood with the VFC cluster with the lowest DHA likelihood, between K13^C580^ rings (E), K13^580Y^ rings (F), K13^C580^ trophozoites (G) and K13^580Y^ trophozoites (H).

**Supplemental Figure S26. Similar pattern of differentially expressed genes using VFC cluster differential expression compared to stage differential expression at the 4 hour timepoint.** Volcano plots in each panel show the average log2FC on the x axis and the negative log10 of the BH-adjusted p value output from MAST [(14)](https://paperpile.com/c/HvL74X/adFa) on the y axis. Colors denote different levels of significance; non significant genes are in grey, genes with an absolute average log2FC > 1 are green, genes with a BH-adjusted p value < 0.001 are in blue and significant genes with an absolute log2FC > 1 and a BH-adjusted p value < 0.001 are in red. The first row represents differential expression results when comparing between DHA vs. DMSO conditions within a stage for the 4 hour timepoint, between K13^C580^ rings (A), K13^580Y^ rings (B), K13^C580^ trophozoites (C) and K13^580Y^ trophozoites (D). The second row represents differential expression results when comparing the VFC cluster with the highest DHA likelihood with the VFC cluster with the lowest DHA likelihood, between K13^C580^ rings (E), K13^580Y^ rings (F), K13^C580^ trophozoites (G) and K13^580Y^ trophozoites (H).

**Supplemental Figure S27. Similar pattern of differentially expressed genes using VFC cluster differential expression compared to stage differential expression at the 6 hour timepoint.** Volcano plots in each panel show the average log2FC on the x axis and the negative log10 of the BH-adjusted p value output from MAST [(14)](https://paperpile.com/c/HvL74X/adFa) on the y axis. Colors denote different levels of significance; non significant genes are in grey, genes with an absolute average log2FC > 1 are green, genes with a BH-adjusted p value < 0.001 are in blue and significant genes with an absolute log2FC > 1 and a BH-adjusted p value < 0.001 are in red. The first row represents differential expression results when comparing between DHA vs. DMSO conditions within a stage for the 6 hour timepoint, between K13^C580^ rings (A), K13^580Y^ rings (B), K13^C580^ trophozoites (C) and K13^580Y^ trophozoites (D). The second row represents differential expression results when comparing the VFC cluster with the highest DHA likelihood with the VFC cluster with the lowest DHA likelihood, between K13^C580^ rings (E), K13^580Y^ rings (F), K13^C580^ trophozoites (G) and K13^580Y^ trophozoites (H).

**Supplemental Table S1 - Counts of Each Annotated Stage per Strain, Timepoint, Treatment Condition and Stage.** We calculated the number of stages within each strain, timepoint and treatment condition.

**Supplemental Table S2 - Differential Expression Results over All Cell Stages**

See the uploaded Excel file.

**Supplemental Table S3 - Gene Ontology Enrichment over All Cell Stages, By Category**

See the uploaded Excel file.

**Supplemental Table S4 - Gene Ontology Enrichment over All Cell Stages, By Gene**

See the uploaded Excel file.

**Supplemental Table S5. Genotypes of Targeted Drug Resistance Marker Loci Captured with Molecular Inversion Probes.**

See the uploaded Excel file.
